# A longitudinal analysis of brain extracellular free water in HIV infected individuals

**DOI:** 10.1038/s41598-021-87801-y

**Published:** 2021-04-15

**Authors:** Md Nasir Uddin, Abrar Faiyaz, Lu Wang, Yuchuan Zhuang, Kyle D. Murray, Maxime Descoteaux, Madalina E. Tivarus, Miriam T. Weber, Jianhui Zhong, Xing Qiu, Giovanni Schifitto

**Affiliations:** 1 Department of Neurology, University of Rochester, Rochester, NY USA; 2 Department of Electrical and Computer Engineering, University of Rochester, Rochester, NY USA; 3 Department of Biostatistics and Computational Biology, University of Rochester, Rochester, NY USA; 4 Department of Physics and Astronomy, University of Rochester, Rochester, NY USA; 5 Department of Computer Science, Université de Sherbrooke, Sherbrooke, QC Canada; 6 Department of Imaging Sciences, University of Rochester, Rochester, NY USA; 7 Department of Neuroscience, University of Rochester, Rochester, NY USA

**Keywords:** Neurology, Neuroscience, Diseases of the nervous system, Image processing, Diagnostic markers, Infectious diseases, HIV infections, Diagnostic markers

## Abstract

Initiation of combination antiretroviral therapy (cART) reduces inflammation in HIV-infected (HIV+) individuals. Recent studies demonstrated that diffusion MRI based extracellular free water (FW) modeling can be sensitive to neuroinflammation. Here, we investigate the FW in HIV-infection, its temporal evolution, and its association with blood markers, and cognitive scores. Using 96 age-matched participants, we found that FW was significantly elevated in grey and white matter in cART-naïve HIV+ compared to HIV-uninfected (HIV−) individuals at baseline. These increased FW values positively correlated with neurofilament light chain (NfL) and negatively correlated with CD4 counts. FW in grey and white matter, as well as NfL decreased in the HIV+ after 12 weeks of cART treatment. No significant FW differences were noted between the HIV+ and HIV− cohorts at 1 and 2-year follow-up. Results suggest that FW elevation in cART-naïve HIV+ participants is likely due to neuroinflammation. The correlation between FW and NfL, and the improvement in both FW and NfL after 12 weeks of cART treatment further reinforces this conclusion. The longer follow-up at 1 and 2 years suggests that cART helped control neuroinflammation as inferred by FW. Therefore, FW could be used as a biomarker to monitor HIV-associated neuroinflammation.

## Introduction

Despite the successful suppression of viral replication and improved immune function with combination antiretroviral therapy (cART), approximately 30–50% of HIV-infected individuals (HIV+) still develop HIV-associated neurological disorders, including cognitive impairment^1^. There is evidence that inflammation persists despite undetectable viral loads; this is thought to be the primary mechanism behind HIV’s disease progression, chronicity and comorbidities such as HIV-associated cognitive impairment^1,2^. Microglia and perivascular macrophages are the primary actors of neuroinflammation^3^ and the primary source of productive HIV infection^4,5^. Quantifying the presence of HIV brain infection, neuroinflammation and neuronal injury associated with the infection are key factors in evaluating the HIV reservoir, the burden of neuroinflammation and the consequences of such neuroinflammation. Evaluation of HIV viral burden in the brain is currently limited to indirect measurements such as those performed using cerebrospinal fluid (CSF). However, CSF viral load is undetectable in > 90% of well-treated individuals with undetectable plasma viral load, thus, CSF viral load may not adequately represent the extent of intra-parenchymal infection^6^.

To date, neuroinflammation due to HIV infection has been assessed using several neuroimaging biomarkers, including elevated glial markers via magnetic resonance spectroscopy (MRS)^7,8^, increased glucose metabolism via Positron Emission Tomography (PET) and increased microglial activation via translocator protein (TSPO) PET tracers^9–11^. While PET ligands offer a more direct quantification of microglia activation^12^, there are several drawbacks. PET imaging is expensive, it exposes individuals to significant ionizing radiation that could limit frequent scanning, and ligands are not always widely available. Further, in cART treated individuals, the expected level of activated microglia is small and the signal captured does not provide information of the effect on neurons^13^. In contrast, the FW index is an emerging diffusion MRI metric that indirectly and noninvasively measures the leakage of extracellular fluid into brain regions. The FW index is associated with neuroinflammatory responses, atrophy, vascular risk factors and cognitive declines^7,13–15^. While the exact interpretation of the FW index is still unclear, one hypothesis is that excessive extracellular FW reflects leakage of serum proteins through the blood brain barrier (BBB) with leaking serum proteins that cause axonal damage and demyelination. It is well established that the disruption of the BBB plays a crucial role in the pathogenesis of HIV-infection^16^.

Free water (FW) imaging, a diffusion MRI post-processing method, has been used to differentiate extracellular non-flowing free water from water that diffuses in proximity to the axons^17^. The FW index, a measure of the relative fraction of freely diffusing water in the extracellular space (ECS), can reveal neuroaxonal damage that affects tissue diffusion characteristics. FW has been found to correlate with neuroinflammation and neurodegeneration in several neurological disorders^18–22^. The relevance of FW as a marker of inflammation has been established by correlating FW with levels of microglial activation measured by TSPO via a PET study^23^. Furthermore, previous animal studies^24–26^ have shown that neuroinflammation alters brain ECS. Previous work by Lo et al.^24^ showed elevated ECS volume in the neocortex of rats inoculated with a strain of Staphylococcus aureus. Di Biase et al.^26^ recently reported increased FW in the frontal WM in adult rats prenatally exposed to maternal immune activation (i.e., exposed to polyriboinosinic-polyribocytidylic acid, Poly-I:C). Another recent work demonstrated elevated FW in WM in mice treated with interferon-gamma (Inf- γ) compared to controls^25^.

The main goals of this work were to determine if FW index is elevated in cART naïve HIV-infected individuals and if it declines with cART treatment, thus providing a marker of responsiveness to treatment. We also posited that if the FW index was indicative of inflammation, it would be correlated with other markers of brain injury such as neurofilament light chain (NfL) and markers of immune function such as CD4 count.

## Methods and materials

### Participants

Forty-four treatment-naïve HIV+ participants (4 females and 40 males; mean age ± standard error, SE = 34.48 ± 1.95 years, range 20–63 years) and 52 age-matched HIV− healthy controls (26 females and 26 males; mean age ± SE = 37.02 ± 1.66 years, range, 18–63 years) were enrolled and followed between 2013 and 2019. The study was reviewed and approved by the Research Subjects Review Board (RSRB) at the University of Rochester. All participants provided written informed consent before enrollment and then underwent clinical, laboratory, neurocognitive and brain MRI exams. All experiments were carried out in accordance with relevant guidelines and regulations. HIV+ participants were evaluated at baseline prior to initiation of cART and at three follow-up time points [12 weeks (n = 38), 1 year (n = 29), and 2 years (n = 19)] post-cART treatment, while the HIV− participants were evaluated at the baseline, 1-year (n = 46), and 2-year (n = 19) time points. The 12-week evaluation of the HIV− participants were not included in the study design as significant changes in MRI and clinical measures in healthy controls are not expected within such a short period. Detailed baseline demographics are presented in Table 1.

**Table 1:** Baseline demographics.

Characteristics	HIV+ (n = 44)	HIV− (n = 52)	p-value
**Age, mean (SE)**	34.48 (1.95)	37.02 (1.66)	0.324
**Sex, n (%)**			** < 0.001**
Female	4 (9.1%)	26 (50%)	
Male	40 (90.9%)	26 (50%)	
**Ethnicity, n (%)**			0.408
Hispanic or Latino	4 (9.1%)	2 (3.8%)	
Not Hispanic or Latino	40 (90.9%)	50 (96.2%)	
**Race, n (%)**			** < 0.001**
Caucasian	20 (45.5%)	42 (80.8%)	
Black AA	22 (50%)	5 (9.6%)	
Other	1 (2.3%)	4 (7.7%)	
Missing	1 (2.3%)	1 (1.9%)	
**Education, n (%)**			**0.039**
≤ 12 years	13 (29.5%)	6 (11.5%)	
> 12 years	31 (70.5%)	46 (88.5%)	
**HAND classification, n (%)**			
Normal	21 (47.7%)	NA	NA
ANI	22 (50.0%)	NA	
MND	1 (2.3%)	NA	

Continuous variables are summarized as Mean ± Standard Error, categorical variables are summarized as frequency (percentages).

Significant p-values are shown as bold.

*HAND* HIV-associated neurocognitive disorders; *ANI* asymptomatic neurocognitive impairment; *MND* mild neurocognitive disorder; *NA* not applicable; *AA* African American.

Inclusion criteria of HIV+ participants included being at least 18 of age, being cART-naïve (both Non-Nucleoside Reverse Transcriptase Inhibitors (NNRTIs) and non NNRTIs based), HIV-1 infection as documented by an HIV test or 2 HIV-1RNA values > 2000 copies/mL at a CLIA certified lab. Further participants fulfilled the following laboratory parameters within 60 days of baseline assessment: hemoglobin ≥ 9.0 g/dL, serum creatinine ≤ 2 × ULN, AST (SGOT), ALT (SGPT), and alkaline phosphatase ≤ 2 × upper limit of normal. Exclusion criteria included severe premorbid or comorbid psychiatric disorders, history of stroke, head trauma resulting in loss of consciousness > 30 min, multiple sclerosis, brain infections (except for HIV-1), and any space-occupying brain lesions requiring acute or chronic therapy. Participants with mild or stable depression including those on stable antidepressant therapy were eligible for this study. Participants were also instructed to avoid smoking and drinking caffeinated beverages at least 2 h prior to each scheduled MRI.

### Data acquisition

#### Blood sample

Plasma levels of neurofilament light chain (NfL) were measured by Simoa assay via a commercial lab (https://www.quanterix.com/, Quanterix, Lexington, MA, United States). Single molecule array (Simoa) by Quanterix is a highly sensitive, single molecule immunoassay, also known as digital enzyme-linked immunosorbent assay. The assay is based upon the capture of low abundance proteins via specific antibodies coated onto paramagnetic dye-coded beads^27^. The quantitative determination of NfL in plasma has a limit of detection of 0.0552 pg/mL (range 0.0152–0.108 pg/mL). HIV viral load (VL) was measured via Roche COBAS 8800 System with a lower limit of detection of 20 copies/mL. CD4 + T lymphocyte count was performed via flow cytometric immunophenotyping at the University of Rochester CLIA (Clinical Laboratory Improvement Amendments) certified clinical lab.

#### Neuropsychological assessments

Trained staff supervised by a licensed clinical neuropsychologist administered the following cognitive battery at each visit for all participants: tests of executive function (Trailmaking Test Parts A & B, Stroop Interference task), speed of information processing (Symbol Digit Modalities Test and Stroop Color Naming), attention and working memory (CalCAP(CRT4), learning [Rey Auditory Verbal Learning Test (Trials 1–5), Rey Complex Figure Test Immediate Recall], memory (Rey Auditory Verbal Learning Test Delayed Recall, Rey Complex Figure Test Delayed Recall), verbal fluency (Controlled Oral Word Association Test), and motor function (Grooved Pegboard, left and right hand). Premorbid intellectual functioning ability was estimated via WRAT-4 Reading at the baseline visit only. Raw scores for each test were converted to z-scores using test manual norms, though z-scores were cut-off at ± 3 (representing 3 standard deviations, SD above and below the mean). Cognitive domain scores were created by averaging the z-scores of tests within each domain. A summary total cognitive score was created by summing the z-scores of the six cognitive domains measured. HAND diagnoses were determined for each participant according to the Frascati criteria^28^, thus asymptomatic neurocognitive impairment (ANI) was defined as neuropsychological impairment (> 1 SD below the demographically appropriate normative mean) in 2 or more cognitive domains with no functional decline, mild neurocognitive disorder (MND) was defined as neuropsychological impairment in 2 or more cognitive domains with mild functional decline, and HIV-associated dementia (HAD) was defined as moderate neuropsychological impairment (> 2 SD below the demographically appropriate normative mean) in more than 2 cognitive domains, with major functional decline.

#### Magnetic resonance imaging

MRI was performed on 3T scanners (MAGNETOM Trio and PrismaFit, Siemens, Erlangen, Germany). The T1-weighted (T1w) images were acquired using a 3D magnetization prepared rapid acquisition gradient-echo (MPRAGE) sequence with inversion time = 1100 ms, repetition time (TR) = 2530 ms; echo time (TE) = 3.44 ms; flip angle = 7°; field of view (FOV) = 256 × 256; GRAPPA = 2; number of slices = 192; voxel size = 1.0 × 1.0 × 1.0 mm^3^. Diffusion weighted images (DWI) were acquired using a single shot spin echo echo-planar imaging (SE-EPI) sequence with the following scan parameters: 60 diffusion-encoded images (b = 1000 s/mm^2^), 10 non-diffusion weighted reference images (b = 0 s/mm^2^); TR = 8900 ms; TE = 86 ms; FOV = 256 × 256; GRAPPA = 2; number of slices = 70; voxel size = 2.0 × 2.0 × 2.0 mm^3^. In order to correct for EPI distortions, a double-echo gradient echo field map sequence was also acquired (TR = 400 ms; TE = 5.19 ms; FOV = 256 × 256; flip angle = 60°; number of slices = 70; voxel size = 2.0 × 2.0 × 2.0 mm^3^).

### Data analysis

Image analyses were performed using a combination of image processing tools and scripting languages, including FSL (https://fsl.fmrib.ox.ac.uk/fsl/fslwiki/)^29^*,* ANTs (http://stnava.github.io/ANTs/)^30^*,* MATLAB (*version 2018b, The MathWorks, Inc., Natick, Massachusetts, United States*) and Python (*version 3.7.4*).

All MRI images were checked for any severe artifacts such as gross geometric distortion, bulk motion, and signal dropout. DWI images were corrected for eddy current-induced distortion, inter-volume participant motion, and susceptibility-induced distortion using the “topup” and “eddy” tools in FSL^31,32^. A bi-tensor model was used to reconstruct FW maps at each voxel from the DWI data using a previously described algorithm^21^ and the processing was carried out using the Nextflow^33^ pipeline with all software dependencies bundled in a Singularity Container^34^. FW values are in the range of 0 to 1. Values approaching 0 correspond to negligible FW diffusion in the ECS while 1 corresponds to unrestricted FW diffusion (i.e., water in a voxel that diffuses completely freely). T1w images were co-registered to the Montreal Neurologic Institute (MNI) standard space (1 mm) using ANTs^30^. The resulting transformations were applied to the FW maps for each subject. We employed the ComBat algorithm to harmonize data across both scanners (due to the scanner upgrade from Siemens Trio to Siemens Prisma) for the FW index for each region-of-interest (ROI)^35^. An empirical Bayesian framework is used in the ComBat algorithm to estimate additive and multiplicative scanner effects.

The FW values were calculated from whole-brain Grey Matter (GM) and White Matter (WM) using corresponding masks. GM and WM masks were created using FSL FAST^36^. In addition, the Harvard–Oxford (subcortical) and JHU-ICBM (WM tracts) atlases were used to calculate regional averages in standard space (1 mm) in 25 pre-defined ROIs relevant to HIV infection^37,38^ [GP: Globus Pallidus; PUT: Putamen; CN: Caudate Nucleus; TH: Thalamus; Hippo: Hippocampus; Amyg: Amygdala; AccN: Accumbens Nucleus; SCC: Splenium of Corpus Callosum; GCC: Genu of Corpus Callosum; CST: Corticospinal Tract; ATR: Anterior Thalamic Radiation; PTR: Posterior Thalamic Radiation; ALIC: Anterior Limbic Internal Capsule; PLIC: Posterior Limbic Internal Capsule; EC: External Capsule; SLF: Superior Longitudinal Fasciculus; FMaj: Forceps Major; FMin: Forceps Minor; CP: Cerebral Peduncle; ACR: Anterior Corona Radiata; SCR: Superior Corona Radiata; RLIC: Retrolenticular part of Internal Capsule; SFOF: Superior Fronto-Occipital Fasciculus; CG: Cingulum and FX: Fornix. FW values were averaged over bilateral ROIs (except some WM tracts]. FW values were averaged over bilateral ROIs for each participant, except for certain WM tracts. ROI results are provided in supplementary tables.

Statistical analyses were performed in R, version 3.6.2 (R Foundation for Statistical Computing, Vienna, Austria). Group comparisons between two independent groups were conducted by either two-group Welch's unequal variances t-test for continuous variables or Fisher’s exact test for categorical variables. Paired t-tests were used to compare the levels of continuous variables in the HIV+ and HIV− participants between baseline and follow-up visits. Pearson correlation tests were used to test the marginal associations between two continuous variables. A p-value of < 0.05 was considered statistically significant for a single hypothesis testing problem. For inferential problems that involved multiple hypotheses, the Benjamini–Hochberg multiple testing procedure was used to control the false discovery rate (FDR) at the 0.05 level^39^.

#### Linear mixed effects regression modeling

Due to the longitudinal nature of the data, most multivariate analyses performed in this study were based on linear mixed effects regressions (LMER), with per-participant random intercepts to account for serial correlations between multiple time points. Empirical evidence shows that the cART treatment effect was most prominent in the first 12 weeks; its long-term effect was subtler and, in some cases, different from its short-term effect. Therefore, we performed two separate LMER analyses to study the longitudinal associations between covariates (such as HIV status and cART treatment) and the response variables (such as FW index and blood markers).

A *Short-Term Model (STM)* was applied to data collected at the baseline (both HIV+ and HIV− participants) and week-12 (HIV+ participants only) visits. Covariates included HIV status (cohort), short-term cART treatment (visit) and age.

A *Long-Term Model (LTM)* was applied to data collected from the HIV− participants at baseline, the HIV+ participants at 12 weeks (treated as the new baseline time point in the LTM), and all participants the year-1 and year-2 follow-ups. Covariates included HIV status, visit, age and the interaction between HIV status and visit.

For both models, parameters were estimated by the restricted maximum likelihood (REML) criterion and the statistical significance was assessed by the adjusted ANOVA *F*-test and regression t-test provided by the package lmerTest in R^40^.

### Approval for human experiments

All protocols were reviewed and approved by the Research Subjects Review Board (RSRB) at the University of Rochester. All participants signed a written consent form.

## Results

### Participant characteristics

Detailed information about demographic, clinical, neurocognitive and MRI data of the study participants are presented in Tables 1 and 2. Welch’s Two Sample t-test indicates that there is no significant age difference between the HIV− and HIV+ participants (p = 0.324). However, we included age as a covariate in all multivariate regression analyses to control for its remaining confounding effects. Compared to HIV+, HIV− participants had higher education levels (p = 0.039) at baseline.

**Table 2:** Extracellular free water, blood markers and cognitive data at baseline and follow-up visits.

Parameter	BSL		W12		Y1		Y2	
	HIV+	HIV−	HIV+	HIV−	HIV+	HIV−	HIV+	HIV−
Participants (n)	44	52	38	0	29	46	19	41
**FW index (%)**								
GM	17.42 ± 0.44	13.76 ± 0.64	14.41 ± 0.91	–	17.91 ± 0.48	17.72 ± 0.41	18.08 ± 0.59	18.03 ± 0.38
WM	4.82 ± 0.12	4.46 ± 0.12	4.44 ± 0.15	–	4.87 ± 0.15	4.98 ± 0.11	4.86 ± 0.21	4.99 ± 0.11
**Blood markers**								
CD4^+^	509.66 ± 41.24	–	587.00 ± 47.20	–	703.76 ± 58.14	–	787.78 ± 67.95	–
VL (× 10^2^)	782.36 ± 205.85	–	4.31 ± 3.96	–	12.39 ± 12.23	–	0.22 ± 0.08	–
NfL	14.65 ± 3.37	7.32 ± 0.56*	10.04 ± 1.67	–	10.37 ± 2.98	–	10.55 ± 2.62	7.51 ± 0.59^+^
Total cognitive score	− 1.62 ± 0.56	0.21 ± 0.59	− 0.19 ± 0.63	–	− 0.42 ± 0.71	1.64 ± 0.75	− 0.46 ± 0.93	2.41 ± 0.78

Values are Mean ± Standard Error; Units: Free Water, FW in %; Viral Load, VL in copies/mL; CD4 + T cell in count/mm^3^; Neurofilament Light Chain, NfL in pg/mL; grey matter, GM; white matter, WM; —indicates data are not available.

*Indicates n = 15.

^+^Indicates n = 10.

### Baseline FW group comparisons

**Figure 1:**
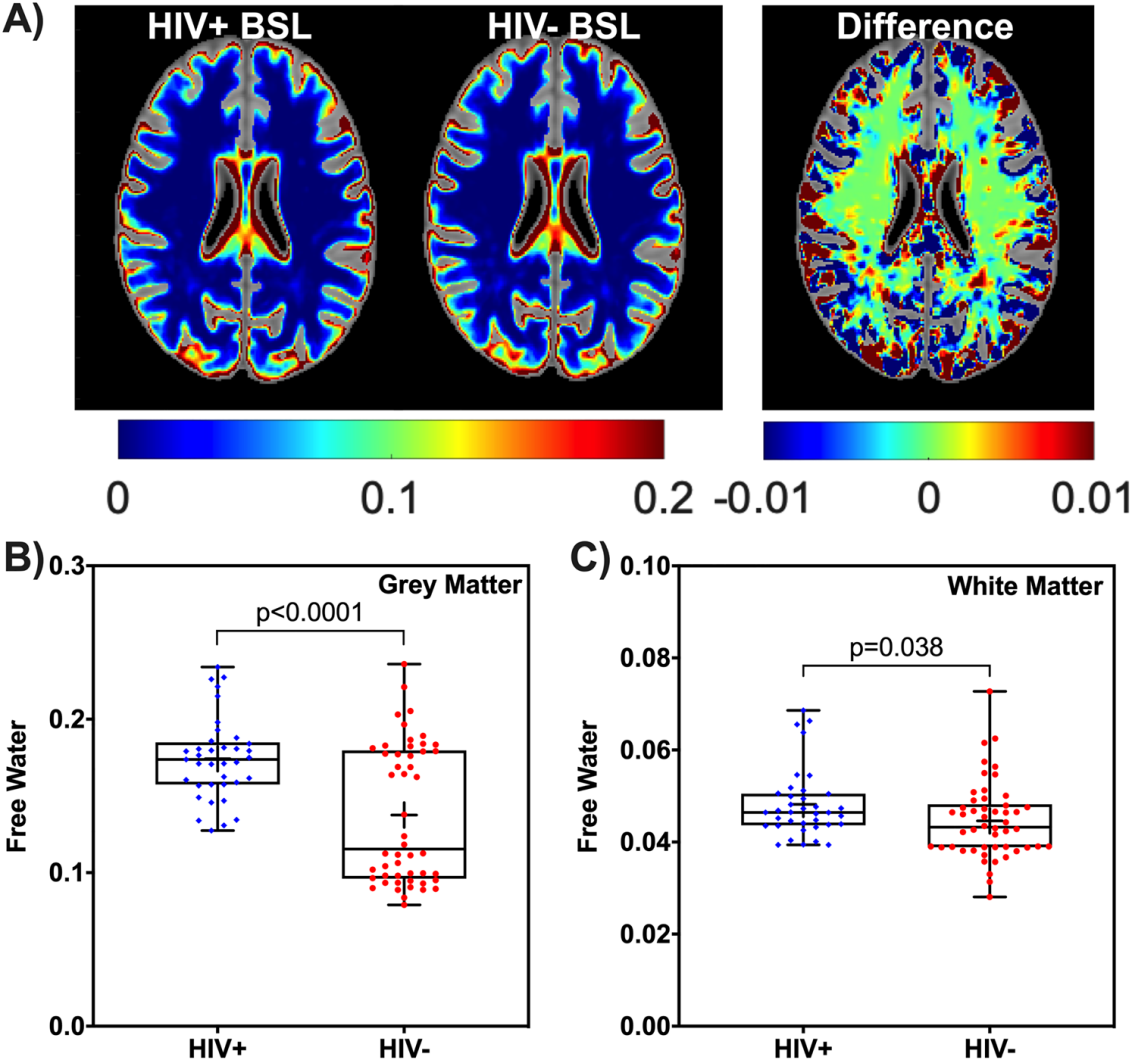
Baseline free water comparison for HIV+ and HIV− cohorts. (**A**) Mean free water (FW) maps (axial view) from 37 HIV+ participants and 52 HIV− participants, and the corresponding difference map at baseline are shown with intensity scales. The box-and-whisker plots illustrate the comparison of the FW index between the HIV+ and HIV− at baseline for (**B**) grey matter (GM) and (**C**) white matter (WM); *Note* significant differences are shown; + sign in the box plots indicates mean FW of each cohort. BSL, Baseline.

Figure 1A shows mean voxel-wise FW maps (axial view) from the HIV+ and HIV− cohorts at baseline and the corresponding difference map. Comparisons of FW between the HIV+ and HIV− groups at baseline for GM and WM are shown in Fig. 1B,C. Group comparisons showed that FW index was higher in GM (t = 4.74, adjusted p-value p_adj_ < 0.001) and in WM (t = 2.11, p_adj_ = 0.038) in the HIV+ cohort (Fig. 1).

Given the relatively large age span of participants, we dichotomized the participants into young adults (age ≤ 30, 22 HIV+ , 18 HIV− participants) and older adults (age > 30, 15 HIV+, 32 HIV− participants), and then compared FW across cohorts. In young adults, FW was significantly higher in GM (t = 10.51, p_adj_ < 0.001) and WM (t = 4.072, p_adj_ < 0.001) in the HIV+ group than the HIV− group. In contrast, in older adults, FW was significantly higher in GM (t = 2.59, p_adj_ = 0.007), only in the HIV+ group compared to the HIV− group.

Among 25 ROIs (See Supplementary Table S1) that included subcortical GM structures and WM tracts, only 3 ROIs (Thalamus, Amygdala and Hippocampus) showed significantly higher FW in the HIV+ cohort than the HIV− cohort (p < 0.05).

We further examined FW relationships by dichotomizing participants as cognitively normal or having cognitive impairment (CI, defined by ANI or MND) within each HIV cohort, independent of age. FW is significantly higher in GM in the HIV+ cohort than HIV− cohort (Normal: t = 3.17, p = 0.003; CI: t = 3.08, p = 0.004). However, we did not find any significant FW differences in GM (t = 1.38, p = 0.176) and WM (t = 0.66, p = 0.517) for normal vs. CI in HIV+ participants.

### Baseline group comparisons of NfL and cognitive performance

In the HIV+ cohort, average NfL concentration was higher (t = 2.10, p = 0.042) while the total cognitive score was lower (t = 1.98, p = 0.028) compared to the HIV− cohort.

### Short term effects of cART on FW

**Figure 2:**
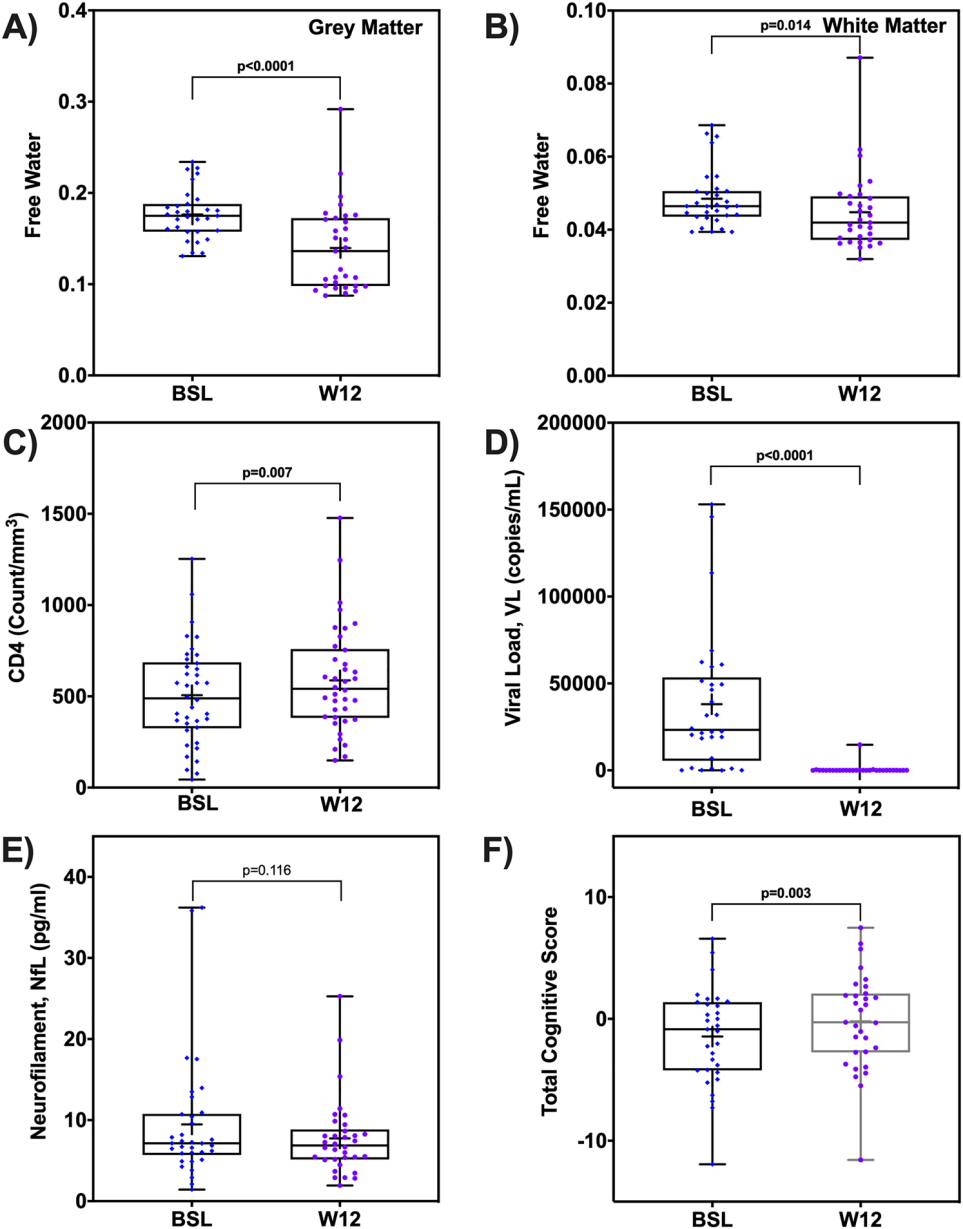
Short term effect of cART treatment on free water, blood markers, and cognitive scores. The box-and-whisker plots illustrate the comparison of free water (FW) index in (**A**) grey matter (GM) and (**B**) white matter (WM); (**C**) CD4 + T cell counts, (**D**) Viral Load (VL), (**D**) average neurofilament light chain (NfL), and E) Total cognitive score in the HIV+ participants. Note: p-values are shown; + sign indicates mean of each measure at each time point. BSL, baseline; W12, week-12.

Comparing baseline to week-12 in HIV+ participants (n = 31 with measures at both baseline and week-12), we found that the FW index decreased significantly in GM (t = 4.57, p_adj_ < 0.001) and WM (t = 2.60, p_adj_ = 0.014) after 12 weeks of cART treatment (Fig. 2A,B). This was also reflected in ROI based analyses (Supplementary Table S2).

Based on the STM that modeled the cohort and treatment effect simultaneously and adjusted for the confounding effects of age, we found that GM and WM are associated with significantly higher FW in the HIV+ cohort (β_cohort_,_GM_ = 0.044, p_adj,GM_ < 0.001; (β_cohort_,_WM_ = 0.005, p_adj,WM_ = 0.004). Even after accounting for the effects of HIV and treatment, age was significantly associated with the increase of FW for most of the ROIs. More information on individual ROIs can be found in Supplementary Table S3.

### Short term effects of cART on blood markers and cognitive performance

CD4 counts increased (t = 2.86, p = 0.007) while VL and the average NfL concentration decreased (VL: t = 3.39, p = 0.002; NfL: t = 1.61, p = 0.115) after 12 weeks of cART treatment (Fig. 2C). Additionally, the total cognitive score increased after 12 weeks of cART treatment in the HIV+ cohort (t = 3.27, p = 0.003) (Fig. 2D).

### Long term effects of cART on FW

**Figure 3:**
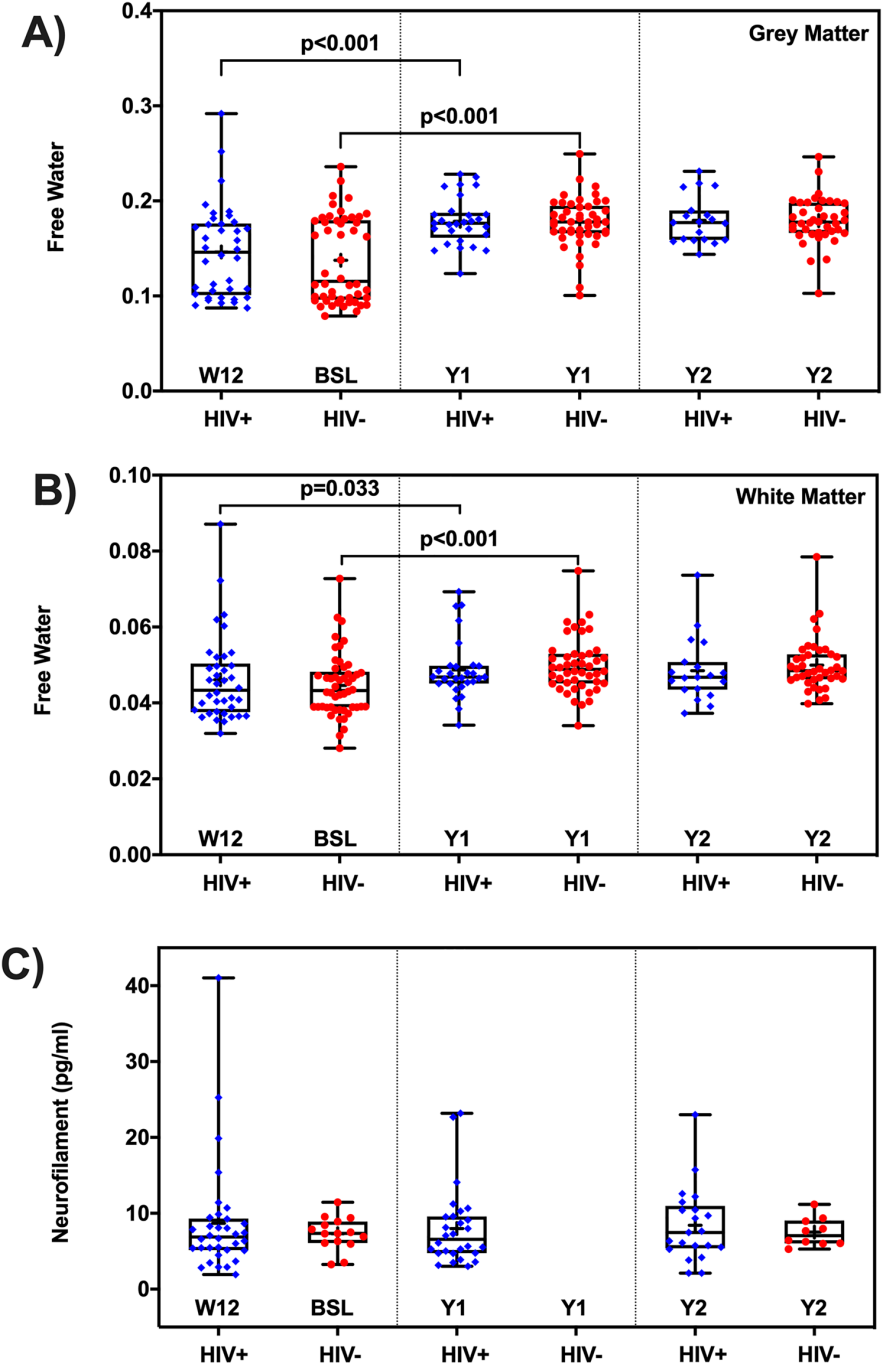
Long term effect of cART treatment on free water and neurofilament light chain. The box-and-whisker plots illustrate the comparison of free water (FW) index between the HIV+ and HIV- participants, and the temporal trends of FW from baseline and follow-up visits in (**A**) gray matter (GM), (**B**) white matter (WM); (**C**) average NfL concentration. There is no significant between-cohort difference of long-term temporal progression in FW (in GM and WM), and NfL. Note, BSL, baseline; W12, week-12 (new BSL for HIV+); Y1, year-1; Y2, year-2; NfL data were not collected at Y1 for HIV− participants. + sign indicates the mean FW at each time point and cohort. Significant differences are also shown.

FW increased in WM and GM at year-1 compared to week-12 and baseline for the HIV+ and HIV− cohorts (p < 0.05). At the year-1 and year-2 visits, there were no significant differences in GM and WM between the HIV+ and HIV− groups (Fig. 3A,B).

The LTM showed there were no significant cohort effects, nor was the interaction of cohort and visit significant in GM and WM, suggesting that levels of FW in the HIV+ cohort were stabilized after 12 weeks of cART treatment (Fig. 3A,B). ROI LTMs revealed similar results and are presented in Supplementary Table S4. Advancing age is associated with a significant increase in FW.

### Long term effects of cART HIV+ on NfL and cognitive performance

Average NfL concentrations stabilized after 12 weeks (new baseline) of cART treatment in the HIV+ cohort. Similar to FW, after 12 weeks of cART treatment, no significant differences in NfL were found between the HIV+ and HIV− cohorts during the follow-up visits (Fig. 3C). Similarly, after 12 weeks of cART treatment, total cognitive scores in the HIV+ cohort stabilized. The cognitive performance improved over time in HIV− individuals likely reflect practice effects, while HIV+ didn’t seem to benefit from practice after 12-weeks and follow-up visits.

### FW association with blood markers and cognitive performance

**Figure 4:**
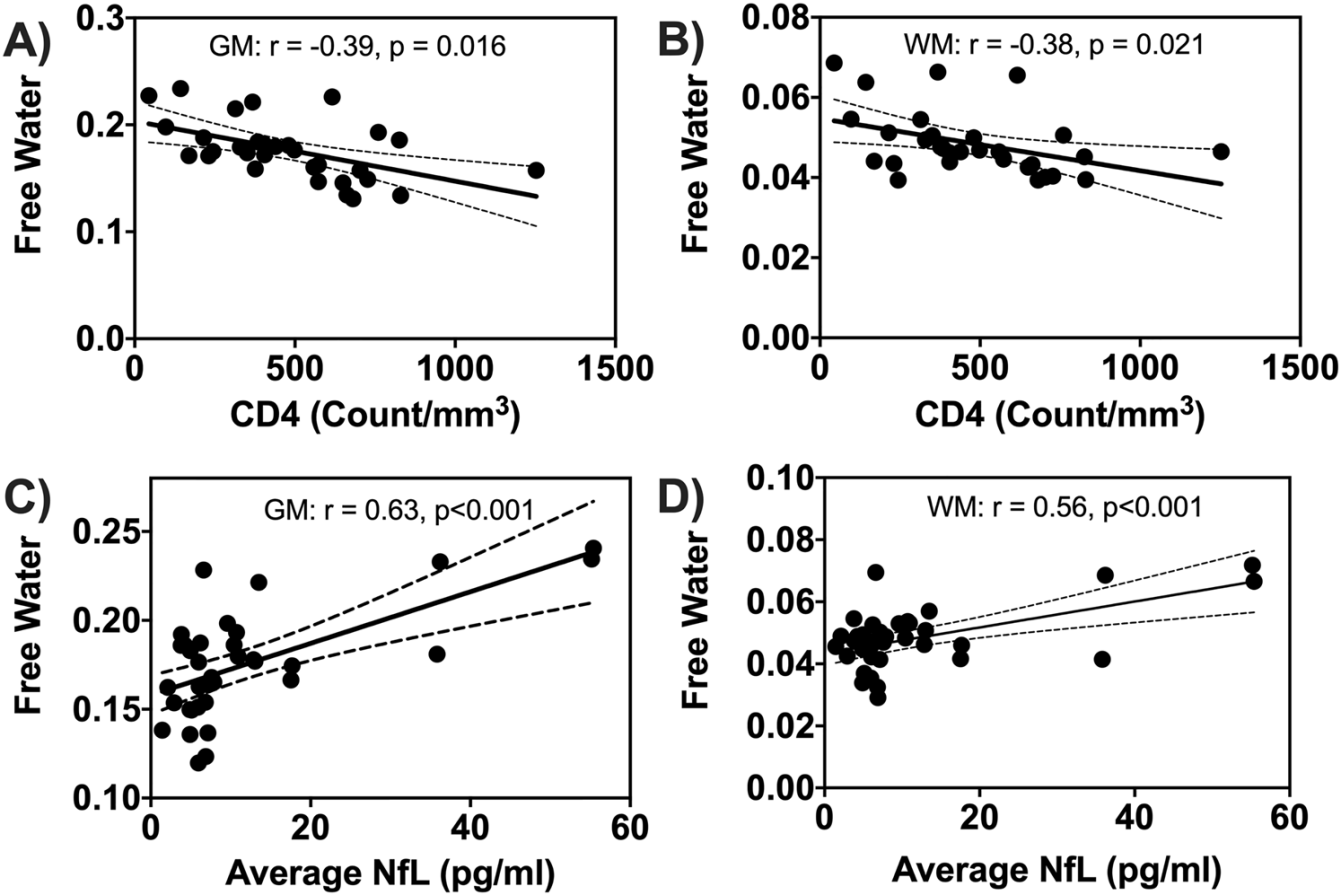
Baseline correlations between free water and blood markers. Scatter plots showing correlations between free water (FW) in grey matter (GM) and white matter (WM) with CD4 counts (**A**,**B)**, and average neurofilament light chain (NfL) **(C**,**D)**. Solid black lines indicate linear fit and dashed lines are 95% confidence interval.

Figure 4 presents the correlations between FW and CD4 counts and average NfL concentrations at baseline. The CD4 cell counts were negatively associated with FW in GM and WM (r ≈ −  0.4, p_adj_ = 0.041), while average NfL concentrations were positively correlated with FW in GM and WM (r ≈ 0.6, p_adj_ < 0.001). FW was positively correlated with NfL in 19 of the 25 ROIs while the CD4 cell counts were negatively associated with FW in 14 of the 25 ROIs. However, no significant correlations were found between FW and VL for any ROI. Details are provided in Supplementary Table S5. VL and FW were positively correlated only in GM at year-1 (r = − 0.42, p = 0.023).

The total cognitive score was unrelated to FW for WM and GM in the HIV− cohort at baseline. Similarly, no associations were found between total cognitive score and FW for WM and GM in both cohorts after grouping by cognitive status (normal and CI). FW for WM and GM was negatively correlated with the total cognitive score in the HIV+ cohort at baseline, but this did not reach statistical significance.

## Discussion

In this study, we used the FW index to indirectly assess neuroinflammation associated with HIV infection. The rationale to use FW as a putative marker of inflammation is based on previous studies in other brain diseases such as PD, AD, and Schizophrenia^18–21,23,26^. We reasoned that HIV-infected cART naïve participants would be at higher risk of inflammation (higher FW) and that treatment would reduce inflammation (lower FW). We also hypothesized that active neuroinflammation would be associated with higher plasma levels of NfL and lower cognitive performance. We further expected that neuroinflammation and thus FW levels to remain reduced to effective cART treatment. Four main findings emerged from this work: (1) FW index and NfL were higher in cART naïve HIV+ compared to the HIV− participants at baseline; (2) FW index and NfL decreased dramatically in GM and WM after 12 weeks of cART treatment in the HIV+ ; (3) FW levels were comparable between the HIV+ and HIV− at 1 year and 2 years of follow-up and similar trends were observed in NfL; and (4) baseline measures of FW index in GM and WM in the HIV+ subjects were associated with CD4 counts and NfL concentration.

To the best of our knowledge, this is the first study to investigate FW as a possible biomarker for neuroinflammation in the presence of HIV-infection. Prior work demonstrates the involvement of subcortical GM and WM structures in neuronal damage in HIV+ individuals^7,37,38,41,42^. We found a significantly higher FW index in GM and WM in cART-naïve HIV+ compared to the HIV− participants at baseline, consistent with previous work in other neurological disorders characterized by neuroinflammation^15,18–21,43^. Increased FW in GM and WM in the HIV+ cohort might be related to an abnormal neuroimmune response^44,45^.

The plasma level of the Neurofilament light chain (NfL) is a promising blood marker for neuroaxonal degradation^46^. NfL is considered a direct measure of neuronal damage since it is released into the brain’s ECS following axonal injury and consequently into the CSF and blood^46,47^. Elevated NfL levels are observed in several neurological and neurodegenerative disorders including HIV-infection^43,47–49^. In this work, the average NfL concentration was significantly higher, by ~ 39%, in the HIV+ compared to HIV− participants.

The FW index was found to be negatively correlated with CD4 counts. Several previous studies suggest that low CD4 counts might be linked to brain atrophy including cortical thinning, reductions in GM and WM volumes and ventricular enlargement^50–52^. Thus, low CD4 counts are associated with elevated extracellular FW in brain tissue. These results provide additional evidence that FW may be related to neuroinflammatory processes. However, in the present study, we did not find an association between FW and VL.

A significant finding was that FW decreased drastically in whole GM and WM and several ROIs in the brain after 12 weeks of cART treatment. FW was decreased significantly by 21% and 8% in the GM and WM respectively by cART treatment. After 12 weeks of cART treatment, FW values in HIV+ participants were close to those of the HIV− participants implying that short term cART treatment normalized FW in the HIV+ cohort. In contrast, the average NfL concentration was reduced by ~ 17% after 12 weeks of cART treatment in the HIV+ cohort. A previous study also reported that NfL levels decreased to normal levels after 6 months of cART initiation in acute HIV-infection^53^. These findings suggest that the change in neuroinflammation due to the treatment can be estimated using the FW index.

After the initial changes due to 12-weeks of cART treatment, no significant differences in FW were observed between the HIV+ and HIV− participants during the two years of follow-up. However, we found that FW increased significantly at year-1 compared to that of baseline in the HIV− participants and compared to the new baseline (post 12 weeks of cART treatment) in the HIV+ participants. Comparison of young adults and older adults revealed that these differences in FW were driven more by the young adults (age < 30 years) in both cohorts. It is not clear whether a yearly change in FW water should be expected as both of our cohorts showed no further changes at year-2 of follow-up. However, it is worth noting that our sample size was significantly lower at year-2 follow-up than year-1. Further investigation with a larger sample is required to confirm these findings. Overall, FW and NfL exhibited similar temporal trends over 2 years of follow-up visits, suggesting that after cART initiation there is likely a minimal level of neuroinflammation.

Of notice, there was no significant correlation between cognitive performance and FW. It is possible that changes in cognitive performance require some neuronal and glia structural damage while FW dynamics can happen at any stage including those where cellular integrity is minimally altered. The trend observed in a negative correlation between cognitive performance and FW may also suggest that the relatively small sample size may have contributed to the limited association. Further investigation is warranted.

This study has a few limitations worth considering. First, the cohorts differed in the proportion of male and female participants, which may have influenced our findings. However, the FW index in GM and WM was not significantly different in males vs. females in our HIV− participants (Supplementary Fig. S2), which had a more equal representation. Second, the number of participants was lower for follow-up visits, especially at year-2 and blood markers were not collected for all participants. Third, FW estimation might be biased by the effects of T2-relaxation as well as blood perfusion. However, a standard approach was used to calculate FW from diffusion MRI data.

## Conclusions

In summary, our findings suggest that extracellular FW is elevated in the brains of cART naïve HIV+ participants at a time when neuroinflammation is expected to be high. Higher levels of NfL and low CD4 counts and their correlations with FW tend to support this possibility. Most importantly, short term cART treatment effectively reduces the levels of FW and stabilizes it over 2 years of follow-up. Although further longitudinal studies with a larger sample size are needed to replicate our findings, FW may represent a potential neuroinflammatory marker that could be used to monitor disease course and response to interventions in HIV-infected individuals.

## Supplementary Information

Supplementary Information.

## Data Availability

Anonymized data will be made available on reasonable request, pending appropriate institutional review board approvals.

## Abbreviations

FW: Free water
FWI: Free water imaging
NfL: Neurofilament light chain
cART: Combination antiretroviral therapy
DTI: Diffusion tensor imaging
GM: Grey matter
WM: White matter
ROI: Region of interest
TSPO: Translocator protein
PET: Positron emission tomography
ECS: Extracellular space
VL: Viral load
SE: Standard error
HAND: HIV-associated neurocognitive disorders
MPRAGE: Magnetization prepared rapid acquisition gradient-echo
DWI: Diffusion weighted imaging
RSRB: Research Subjects Review Board
TR: Repetition time
TE: Echo time
FOV: Field of view
FDR: False discovery rate
LMER: Linear mixed effects regressions
STM: Short-term model
LTM: Long-term model
REML: Restricted maximum likelihood
p_adj_: Adjusted p-value
BBB: Blood brain barrier
MRS: Magnetic resonance spectroscopy
