# Supplementary material for: A longitudinal analysis of brain extracellular free water in HIV infected individuals
Source: Sci Rep. 2021 Apr 15;11:8273. doi: 10.1038/s41598-021-87801-y (PMC8050285; doi:10.1038/s41598-021-87801-y)

Supplementary Materials

Title: A Longitudinal Analysis of Brain Extracellular Free Water in HIV Infected Individuals

Authors: Md Nasir Uddin[1,*], Abrar Faiyaz[2], Lu Wang[3], Yuchuan Zhuang[2], Kyle D Murray[4], Maxime Descoteaux[5], Madalina E. Tivarus[6,7], Miriam T. Weber[1], Jianhui Zhong[4,6], Xing Qiu[3], and Giovanni Schifitto[1,6]

Affiliations:
[1]Department of Neurology, University of Rochester, Rochester, NY, USA
[2]Department of Electrical and Computer Engineering, University of Rochester, Rochester, NY, USA
[3]Department of Biostatistics and Computational Biology, University of Rochester, Rochester, NY, USA
[4]Department of Physics and Astronomy, University of Rochester, Rochester, NY, USA
[5]Department of Computer Science, Université de Sherbrooke, QC, Canada
[6]Department of Imaging Sciences, University of Rochester, Rochester, NY, USA
[7]Department of Neuroscience, University of Rochester, Rochester, NY, USA

Table S1: Mean and standard error (SE) of FW index (%) for 25 brain structures in HIV+ and HIV- participants at baseline

**Structure**	**HIV+**		**HIV-**		**t-value**	**p-value**	**p-value (Adjusted)**
	**mean**	**SE**	**mean**	**SE**			
FMaj	7.61	0.35	7.48	0.34	0.276	0.784	0.948
FMin	4.11	0.17	3.79	0.12	1.548	0.126	0.788
GCC	11.34	0.50	10.44	0.39	1.411	0.162	0.812
SCC	9.92	0.30	9.95	0.27	-0.082	0.935	0.948
TH	14.73	0.50	13.27	0.49	2.093	**0.039**	0.328
CN	11.46	0.87	10.5	0.77	0.826	0.412	0.928
PUT	0.65	0.06	0.68	0.05	-0.376	0.708	0.948
GP	0.48	0.07	0.61	0.14	-0.835	0.406	0.928
Amyg	18.30	0.50	15.29	0.5	4.239	**<0.001**	**0.002**
AccN	1.87	0.29	1.79	0.23	0.226	0.822	0.948
ATR	2.83	0.14	2.75	0.13	0.394	0.695	0.948
CST	2.26	0.10	2.34	0.09	-0.652	0.516	0.928
SLF	1.48	0.14	1.46	0.12	0.127	0.900	0.948
ALIC	0.14	0.02	0.16	0.02	-0.661	0.511	0.928
PLIC	0.07	0.01	0.05	0.01	0.906	0.369	0.928
PTR	3.86	0.22	3.83	0.21	0.104	0.917	0.948
EC	0.37	0.04	0.35	0.04	0.213	0.832	0.948
HC	21.5	0.75	19.14	0.61	2.444	**0.017**	0.211
CP	6.97	0.30	6.81	0.27	0.376	0.708	0.948
ACR	0.11	0.03	0.11	0.03	0.066	0.948	0.948
SCR	0.03	0.01	0.04	0.02	-0.232	0.817	0.948
RLIC	0.60	0.08	0.51	0.04	1.119	0.268	0.928
SFOF	0.23	0.08	0.17	0.05	0.648	0.519	0.928
CG	0.76	0.05	0.69	0.05	1.117	0.267	0.928
FX	46.46	1.55	44.68	1.35	0.867	0.389	0.928

Note: Significant p-values are shown as bold. GP: Globus Pallidus; PUT: Putamen; CN: Caudate Nucleus; TH: Thalamus; Hippo: Hippocampus; Amyg: Amygdala; AccN: Accumbens Nucleus; SCC: Splenium of Corpus Callosum; GCC: Genu of Corpus Callosum; CST: Corticospinal Tract; ATR: Anterior Thalamic Radiation; PTR: Posterior Thalamic Radiation; ALIC: Anterior Limbic Internal Capsule; PLIC: Posterior Limbic Internal Capsule; EC: External Capsule; SLF: Superior Longitudinal Fasciculus; FMaj: Forceps Major; FMin: Forceps Minor; CP: Cerebral Peduncle; ACR: Anterior Corona Radiata; SCR: Superior Corona Radiata; RLIC: Retrolenticular part of Internal Capsule; SFOF: Superior Fronto-Occipital Fasciculus; CG: Cingulum and FX: Fornix. FW values were averaged over bilateral ROIs (except some WM tracts) for each participant.

Table S2: Mean and standard error (SE) of FW index (%) for 25 brain structures at baseline and 12 weeks after the cART treatment in HIV+ participants

**Structure**	**BSL**		**W12**		**t-value**	**p-value**	**p-value (Adjusted)**
	**mean**	**SE**	**mean**	**SE**			
FMaj	7.40	0.36	7.00	0.28	1.771	0.087	0.241
Fmin	4.14	0.19	3.80	0.22	2.328	**0.027**	0.096
GCC	11.37	0.56	9.96	0.55	3.417	**0.002**	**0.015**
SCC	9.83	0.33	9.79	0.43	0.155	0.878	0.878
TH	14.9	0.57	13.2	0.68	3.767	**0.001**	**0.009**
CN	11.58	1.00	10.31	0.91	2.776	**0.009**	**0.039**
PUT	0.67	0.07	0.70	0.09	-0.442	0.662	0.790
GP	0.49	0.08	0.52	0.12	-0.248	0.806	0.876
Amyg	18.54	0.56	15.42	0.67	4.865	**<0.001**	**0.001**
AccN	1.95	0.35	1.64	0.25	1.594	0.121	0.304
ATR	2.86	0.16	2.74	0.18	1.388	0.175	0.365
CST	2.26	0.12	2.21	0.13	0.620	0.540	0.790
SLF	1.53	0.16	1.48	0.18	0.672	0.507	0.790
ALIC	0.13	0.02	0.14	0.02	-0.545	0.590	0.790
PLIC	0.06	0.01	0.04	0.01	1.455	0.156	0.355
PTR	3.73	0.23	3.28	0.23	2.971	**0.006**	**0.034**
EC	0.37	0.05	0.36	0.05	0.191	0.849	0.878
HC	22.01	0.85	20.62	0.84	2.902	**0.007**	**0.034**
CP	6.91	0.34	6.84	0.39	0.254	0.801	0.876
ACR	0.11	0.03	0.10	0.02	0.439	0.664	0.790
SCR	0.03	0.01	0.03	0.01	0.458	0.650	0.790
RLIC	0.60	0.09	0.44	0.04	2.031	0.051	0.160
SFOF	0.24	0.1	0.21	0.05	0.525	0.603	0.790
CG	0.74	0.05	0.68	0.06	1.159	0.256	0.491
FX	46.72	1.73	46.2	1.84	0.610	0.547	0.790

Note: Significant p-values are shown as bold. GP: Globus Pallidus; PUT: Putamen; CN: Caudate Nucleus; TH: Thalamus; Hippo: Hippocampus; Amyg: Amygdala; AccN: Accumbens Nucleus; SCC: Splenium of Corpus Callosum; GCC: Genu of Corpus Callosum; CST: Corticospinal Tract; ATR: Anterior Thalamic Radiation; PTR: Posterior Thalamic Radiation; ALIC: Anterior Limbic Internal Capsule; PLIC: Posterior Limbic Internal Capsule; EC: External Capsule; SLF: Superior Longitudinal Fasciculus; FMaj: Forceps Major; FMin: Forceps Minor; CP: Cerebral Peduncle; ACR: Anterior Corona Radiata; SCR: Superior Corona Radiata; RLIC: Retrolenticular part of Internal Capsule; SFOF: Superior Fronto-Occipital Fasciculus; CG: Cingulum and FX: Fornix. FW values were averaged over bilateral ROIs (except some WM tracts) for each participant.

Table S3: Cohort and 12 weeks of cART treatment effects in free water (FW) based on the short-term model (STM) for 25 brain structures.

**Structure**	**ß ×10^{-3}**			**p-value**		
	**Cohort**	**Visit**	**Age**	**Cohort**	**Visit**	**Age**
FMaj	0.30	-4.50	-0.10	0.946	0.052	0.730
Fmin	4.10	-3.70	0.30	0.054	**0.012**	**0.001**
GCC	10.90	-15.20	0.70	0.067	**<0.001**	**0.002**
SCC	1.40	-0.60	0.30	0.738	0.807	**0.048**
TH	20.90	-17.10	1.40	**0.001**	**<0.001**	**<0.001**
CN	22.40	-12.50	2.40	**0.020**	**0.009**	**<0.001**
PUT	0.40	0.20	0.20	0.631	0.660	**<0.001**
GP	-0.40	0.00	0.30	0.815	0.997	**<0.001**
Amyg	37.80	-27.60	1.30	**<0.001**	**<0.001**	**<0.001**
AccN	3.00	-3.40	0.60	0.318	0.085	**<0.001**
ATR	2.50	-1.30	0.40	0.114	0.140	**<0.001**
CST	1.70	0.00	0.20	0.326	0.987	**0.005**
SLF	2.20	-0.50	0.40	0.155	0.532	**<0.001**
ALIC	0.30	0.10	0.10	0.406	0.493	**<0.001**
PLIC	0.30	-0.10	0.00	0.063	0.194	**0.047**
PTR	1.80	-4.40	0.10	0.562	**0.006**	0.467
EC	0.80	0.00	0.10	0.125	0.896	**<0.001**
HC	30.00	-13.60	1.60	**0.001**	**0.007**	**<0.001**
CP	10.00	0.70	0.40	0.083	0.780	0.088
ACR	0.20	-0.20	0.00	0.483	0.506	**<0.001**
SCR	0.00	-0.10	0.00	0.960	0.555	**0.015**
RLIC	1.60	-1.00	0.10	**0.047**	0.174	**0.004**
SFOF	1.00	-0.40	0.10	0.178	0.528	**0.001**
CG	3.40	-0.30	0.20	**0.008**	0.561	**<0.001**
FX	33.00	-6.30	3.90	0.060	0.462	**<0.001**

Note: Significant p-values are shown as bold. GP: Globus Pallidus; PUT: Putamen; CN: Caudate Nucleus; TH: Thalamus; Hippo: Hippocampus; Amyg: Amygdala; AccN: Accumbens Nucleus; SCC: Splenium of Corpus Callosum; GCC: Genu of Corpus Callosum; CST: Corticospinal Tract; ATR: Anterior Thalamic Radiation; PTR: Posterior Thalamic Radiation; ALIC: Anterior Limbic Internal Capsule; PLIC: Posterior Limbic Internal Capsule; EC: External Capsule; SLF: Superior Longitudinal Fasciculus; FMaj: Forceps Major; FMin: Forceps Minor; CP: Cerebral Peduncle; ACR: Anterior Corona Radiata; SCR: Superior Corona Radiata; RLIC: Retrolenticular part of Internal Capsule; SFOF: Superior Fronto-Occipital Fasciculus; CG: Cingulum and FX: Fornix. FW values were averaged over bilateral ROIs (except some WM tracts) for each participant

Table S4: Cohort and long-term cART treatment effects in free water (FW) based on the long-term model (LTM) for 25 brain structures.

**Structure**	**ß ×10^{-3}**				**p-value**			
	**Cohort**	**Visit**	**Age**	**Cohort.Visit**	**Cohort**	**Visit**	**Age**	**Cohort.Visit**
FMaj	-5.80	1.60	0.00	3.30	0.215	0.080	0.911	**0.038**
FMin	0.20	1.00	0.20	-0.80	0.919	0.067	**0.025**	0.426
GCC	-4.20	4.90	0.50	1.70	0.468	**0.003**	**0.014**	0.533
SCC	0.10	2.10	0.30	-0.40	0.973	0.054	0.071	0.820
TH	3.00	7.90	1.30	-0.20	0.628	**<0.001**	**<0.001**	0.952
CN	9.50	9.00	2.30	-0.70	0.352	**<0.001**	**<0.001**	0.833
PUT	0.50	0.00	0.10	0.00	0.591	0.817	**<0.001**	0.899
GP	-0.70	-0.10	0.20	-0.10	0.622	0.894	**<0.001**	0.917
Amyg	7.60	16.60	1.10	-0.80	0.231	**<0.001**	**<0.001**	0.853
AccN	0.00	-1.00	0.50	3.90	0.999	0.373	**<0.001**	**0.034**
ATR	1.00	0.30	0.40	-0.50	0.529	0.423	**<0.001**	0.432
CST	1.50	1.30	0.20	-0.70	0.413	**0.001**	**0.015**	0.273
SLF	1.80	0.80	0.40	-1.20	0.296	**0.009**	**<0.001**	**0.029**
ALIC	0.40	0.50	0.10	-0.30	0.315	**0.001**	**<0.001**	0.349
PLIC	0.20	0.20	0.00	0.00	0.317	**0.008**	**0.044**	0.721
PTR	-2.90	2.10	0.10	1.90	0.375	**0.010**	0.659	0.180
EC	0.70	0.50	0.10	-0.30	0.198	**0.002**	**<0.001**	0.233
HC	16.60	5.50	1.50	0.40	0.077	**<0.001**	**<0.001**	0.862
CP	9.60	3.90	0.20	-4.90	0.086	**0.036**	0.220	0.119
ACR	0.00	0.10	0.00	0.10	0.889	**0.045**	**0.006**	0.593
SCR	-0.10	0.00	0.00	0.00	0.772	0.072	**0.045**	0.991
RLIC	0.90	1.10	0.10	-0.10	0.295	**<0.001**	**0.022**	0.836
SFOF	0.70	0.30	0.10	-0.40	0.278	**0.022**	**0.003**	0.110
CG	3.10	1.50	0.20	-1.50	**0.015**	**<0.001**	**0.001**	**0.029**
FX	23.40	2.70	3.90	-8.60	0.230	0.603	**<0.001**	0.329

Note: Significant p-values are shown as bold. GP: Globus Pallidus; PUT: Putamen; CN: Caudate Nucleus; TH: Thalamus; Hippo: Hippocampus; Amyg: Amygdala; AccN: Accumbens Nucleus; SCC: Splenium of Corpus Callosum; GCC: Genu of Corpus Callosum; CST: Corticospinal Tract; ATR: Anterior Thalamic Radiation; PTR: Posterior Thalamic Radiation; ALIC: Anterior Limbic Internal Capsule; PLIC: Posterior Limbic Internal Capsule; EC: External Capsule; SLF: Superior Longitudinal Fasciculus; FMaj: Forceps Major; FMin: Forceps Minor; CP: Cerebral Peduncle; ACR: Anterior Corona Radiata; SCR: Superior Corona Radiata; RLIC: Retrolenticular part of Internal Capsule; SFOF: Superior Fronto-Occipital Fasciculus; CG: Cingulum and FX: Fornix. FW values were averaged over bilateral ROIs (except some WM tracts) for each participant.

Table S5: Pearson Correlations for FW vs. NfL, FW vs. CD4 counts and FW vs. VL at baseline for 25 brain structures in HIV+ cohort

**Structure**	**NfL**		**CD4 counts**		**VL**	
	**ß**	**p-value**	**ß**	**p-value**	**ß**	**p-value**
FMaj	0.045	0.797	-0.204	0.227	-0.072	0.688
FMin	0.479	**0.004**	-0.314	0.058	0.183	0.301
GCC	0.462	**0.005**	-0.256	0.127	0.100	0.575
SCC	0.438	**0.008**	-0.334	**0.043**	0.029	0.872
TH	0.621	**<0.001**	-0.351	**0.033**	-0.042	0.812
CN	0.503	**0.002**	-0.372	**0.023**	-0.104	0.558
PUT	0.415	**0.013**	-0.434	**0.007**	0.022	0.902
GP	0.366	**0.031**	-0.428	**0.008**	-0.058	0.746
Amyg	0.656	**<0.001**	-0.318	0.055	0.056	0.751
AccN	0.564	**<0.001**	-0.390	**0.017**	-0.032	0.857
ATR	0.627	**<0.001**	-0.339	**0.040**	-0.075	0.673
CST	0.455	**0.006**	-0.464	**0.004**	-0.105	0.555
SLF	0.474	**0.004**	-0.356	**0.031**	0.057	0.748
ALIC	0.536	**0.001**	-0.368	**0.025**	-0.210	0.233
PLIC	0.023	0.894	-0.076	0.653	-0.198	0.262
PTR	0.174	0.318	-0.101	0.553	-0.004	0.984
EC	0.325	0.057	-0.206	0.222	-0.189	0.285
HC	0.696	**<0.001**	-0.378	**0.021**	0.025	0.890
CP	0.062	0.722	-0.182	0.282	-0.002	0.993
ACR	0.567	**<0.001**	-0.358	**0.030**	0.009	0.959
SCR	0.595	**<0.001**	-0.357	**0.030**	-0.085	0.634
RLIC	0.572	**<0.001**	-0.251	0.134	-0.117	0.510
SFOF	0.495	**0.002**	-0.253	0.130	0.037	0.837
CG	0.214	0.217	-0.234	0.164	-0.184	0.297
FX	0.491	**0.003**	-0.407	**0.012**	-0.104	0.559

Note: Significant p-values are shown as bold. NfL: Neurofilament llight chain; CD4: Cluster of differentiation 4; VL Viral Loads. GP: Globus Pallidus; PUT: Putamen; CN: Caudate Nucleus; TH: Thalamus; Hippo: Hippocampus; Amyg: Amygdala; AccN: Accumbens Nucleus; SCC: Splenium of Corpus Callosum; GCC: Genu of Corpus Callosum; CST: Corticospinal Tract; ATR: Anterior Thalamic Radiation; PTR: Posterior Thalamic Radiation; ALIC: Anterior Limbic Internal Capsule; PLIC: Posterior Limbic Internal Capsule; EC: External Capsule; SLF: Superior Longitudinal Fasciculus; FMaj: Forceps Major; FMin: Forceps Minor; CP: Cerebral Peduncle; ACR: Anterior Corona Radiata; SCR: Superior Corona Radiata; RLIC: Retrolenticular part of Internal Capsule; SFOF: Superior Fronto-Occipital Fasciculus; CG: Cingulum and FX: Fornix. FW values were averaged over bilateral ROIs (except some WM tracts) for each participant.

Supplementary Figures

Figure S1: Free water comparison for HIV+ and HIV- cohorts

Mean free water (FW) maps (axial view) from HIV+ participants and HIV- participants at baseline and follow-up visits (top), and the corresponding difference maps (bottom) are shown with intensity scales. BSL, Baseline; W12, week-12; Y1, year-1; Y2, year-2; BSL-W12, FW difference between baseline and week-12; BSL-Y1, FW difference between baseline and year-2; BSL-Y2, FW difference between baseline and year-2; Y1-Y2, FW difference between year-1 and year-2;

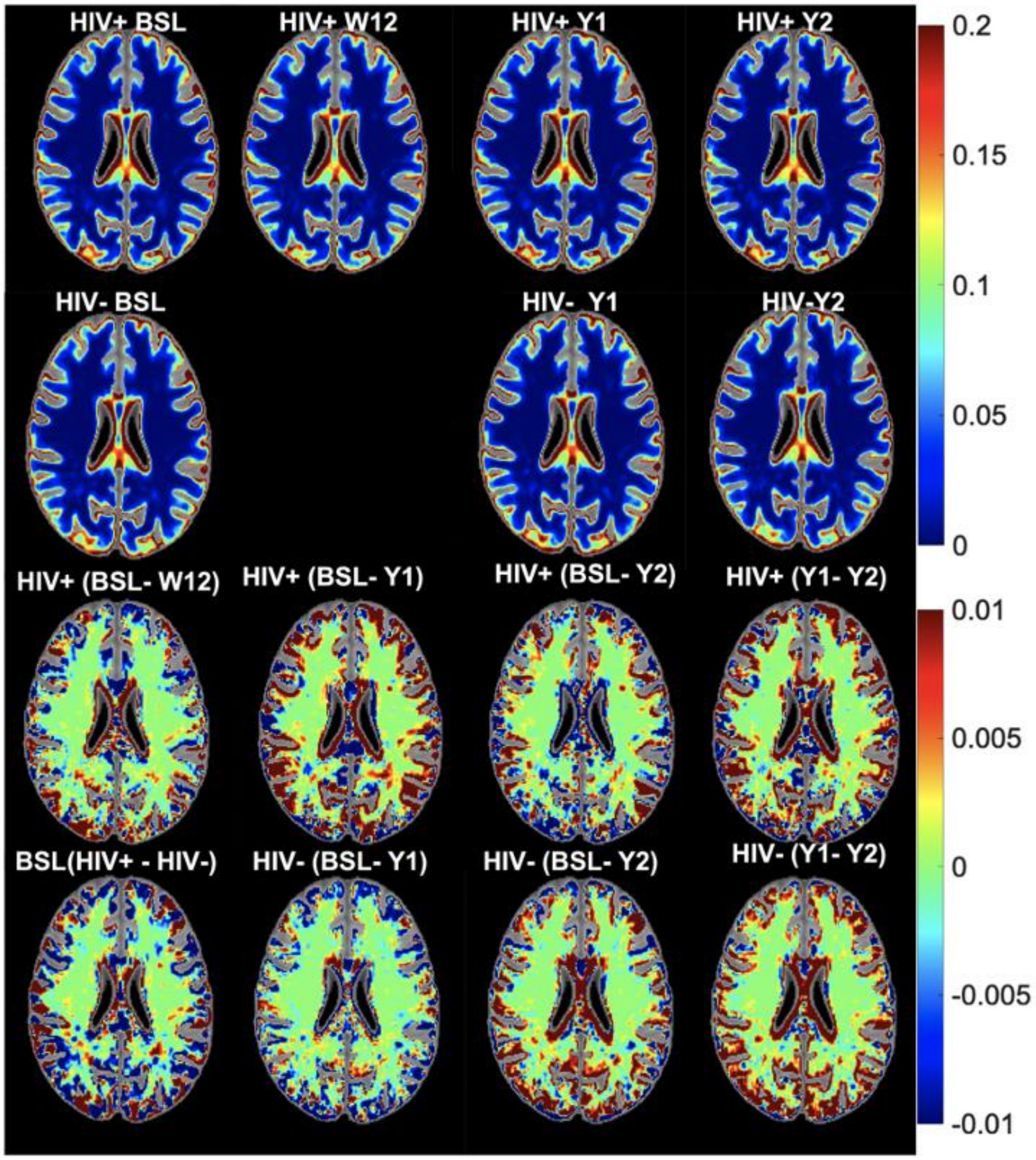

Figure S2: Free water comparison between male and female in HIV- cohort

The box-and-whisker plots illustrate the comparison of free water (FW) index in grey matter (GM) and white matter (WM) between the male and female in HIV- participants. ns, not significant.

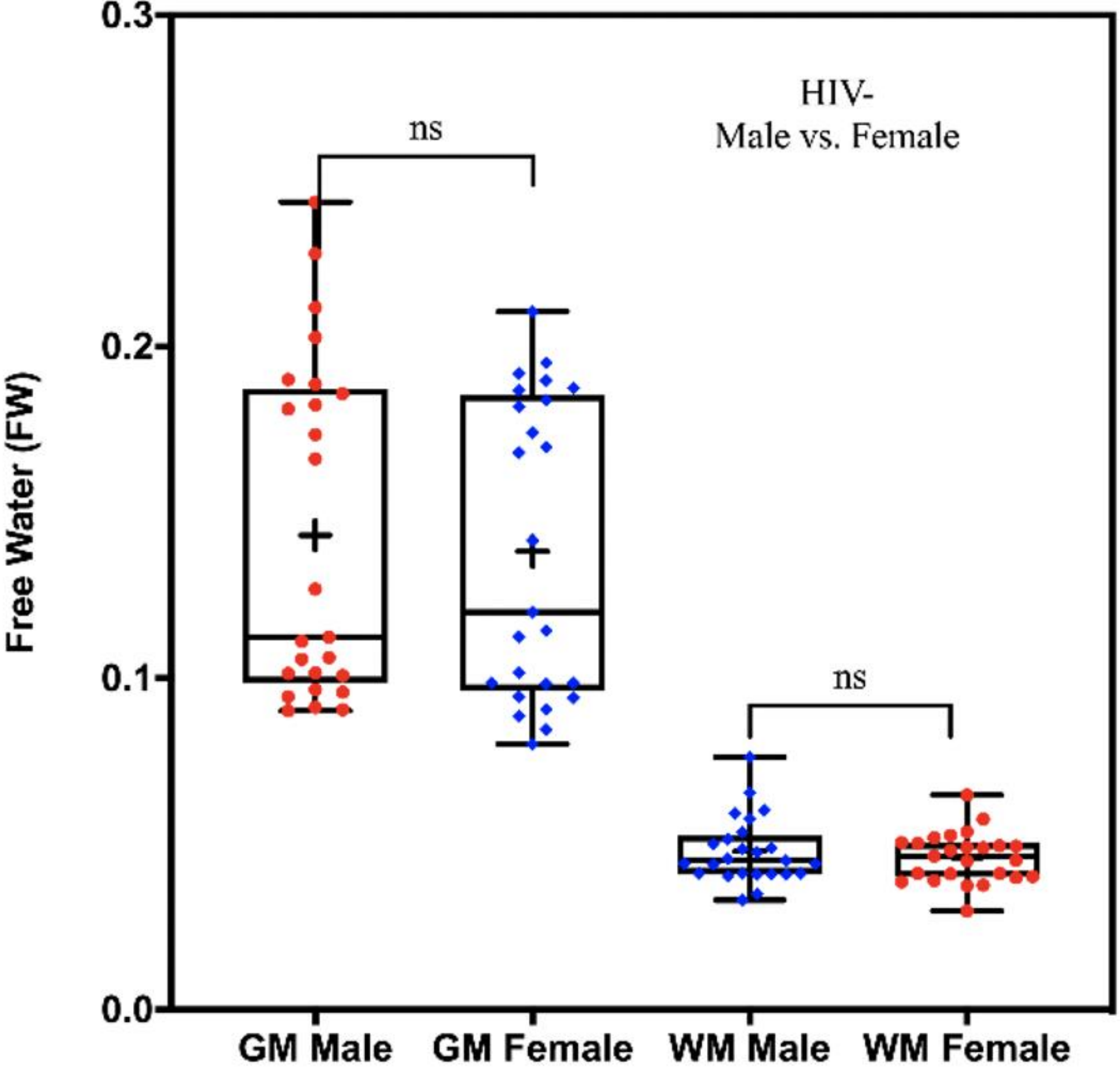

Figure S3: Free water versus age at baseline

Scatter plots between free water (FW) in grey matter (GM) and white matter (WM) with age (combined HIV+ and HIV- cohorts). Solid black lines indicate linear fit, and dashed lines indicate 95% confidence intervals; Pearson r and p-values are also provided.

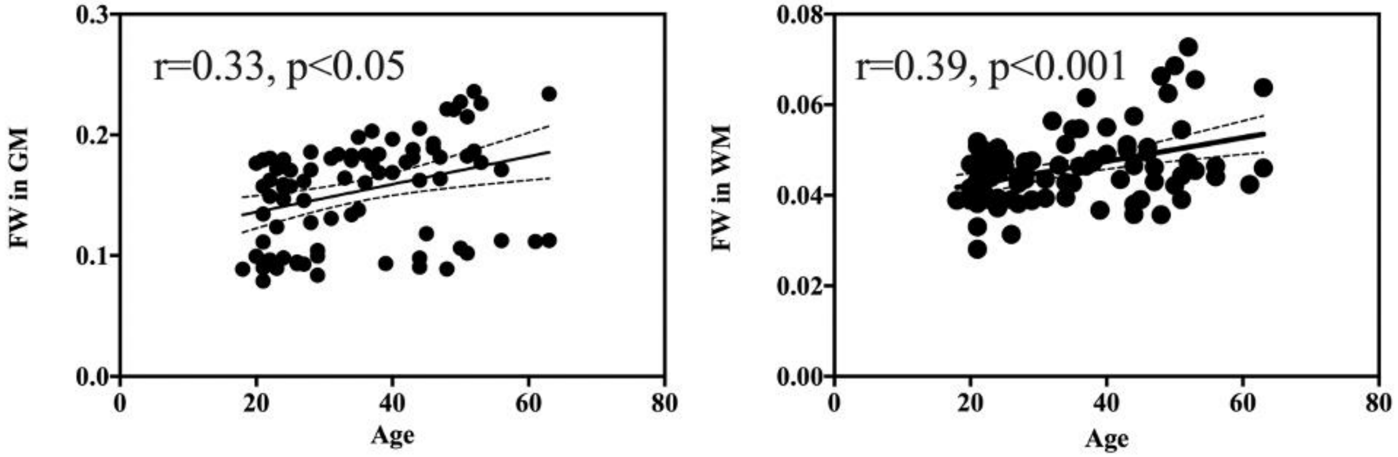

Supplement: Supplementary file 1 — Supplementary Information. [file 41598_2021_87801_MOESM1_ESM.pdf]
